# A qualitative study of therapists’ use of empirically supported techniques for PTSD

**DOI:** 10.3389/fpsyg.2025.1542478

**Published:** 2025-08-01

**Authors:** Erin Neill, Amie Zarling, Carl F. Weems

**Affiliations:** ^1^Department of Human Development and Family Studies, Iowa State University, Ames, IA, United States; ^2^Heart Counseling LLC, Madison, WI, United States

**Keywords:** qualitative, therapist, traumatic, PTSD, CBT, EMDR

## Abstract

**Introduction:**

Understanding how practicing therapists implement and perceive exposure techniques, as well as other empirically supported treatment components for posttraumatic stress disorder (PTSD), such as those found in cognitive-behavioral therapy (CBT) and eye movement desensitization and reprocessing (EMDR), is essential for improving the delivery of effective interventions. This study aims to contribute to that effort by exploring the experiences and attitudes of therapists who treat PTSD in clinical practice.

**Methods:**

As part of a broader mixed-methods inquiry, in-depth interviews were conducted.

**Results:**

Findings aligned with several *a priori* themes, while additional themes also emerged from the data. The findings suggest that therapists often employ a client-centered integration of CBT and EMDR techniques. There was also a noted reluctance to use exposure sessions in CBT for PTSD, particularly when therapists interpreted exposure primarily as *in vivo* exposure. While many interviewees equated exposure techniques with *in vivo* sessions, some viewed EMDR as a form of imaginal exposure.

**Discussion:**

These findings contribute to the growing body of qualitative research on therapist-related factors that influence the implementation of effective PTSD interventions. Additional themes are discussed, along with implications for improving intervention delivery and directions for future research.

## Introduction

Exposure to traumatic and adverse life experiences is strongly associated with the development of posttraumatic stress disorder (PTSD) in a substantial portion of individuals (Bradley et al., 2005; Cohen et al., 2006; Weems et al., 2021). Both cognitive-behavioral therapy (CBT) and eye movement desensitization and reprocessing (EMDR) have empirical support and have been endorsed for the treatment of PTSD by the International Society for Traumatic Stress Studies (see ISTSS, 2018; Balbo et al., 2019). However, deploying and implementing these evidence-based treatments in real-world practice settings remains a challenge (Herschell et al., 2010; Neill et al., 2023; Weems, 2019; Weisz et al., 2015).

Exposure techniques in behavioral therapy typically involve gradually confronting feared situations or objects—either *in vivo* or through imagination—in a controlled setting, combined with fear-reduction strategies and encouragement of non-avoidance. While these are core to many broader intervention models for PTSD, the literature suggests they remain under-utilized in practice settings (Becker et al., 2004; Borntrager et al., 2013; Cook et al., 2004; Farrell et al., 2013; Foy et al., 1996; Harned et al., 2013; Neill et al., 2023). Understanding practicing therapists’ attitudes and implementation of exposure techniques, as well as use and integration of treatment components for PTSD, such as those in CBT and EMDR, may be an avenue to improve the deployment of efficacious interventions to those in need.

At the outset, it is useful to note that his manuscript was guided by the specific components commonly found in effective interventions for PTSD, though treatment protocols with broad modalities have traditionally been the focus of most clinical trials. Chorpita et al. (2005) introduced the distillation and matching model, which matches distilled components from efficacious treatment protocols to client needs based on presenting problems or demographics. For example, Chorpita and Daleiden’s (2009) application of the distillation and matching model identified core elements—such as exposure, cognitive strategies, psychoeducation, and relaxation—as the most frequently used in successful randomized controlled trials (RCTs) for child trauma. Chorpita and Daleiden (2009) identified 41 distinct practice element codes from an analysis of 232 treatment studies, organizing them using a method similar to exploratory factor analysis. In their review of 11 treatments specifically targeting traumatic stress, the most frequently used components were exposure techniques and cognitive strategies (both with a frequency of 0.91), followed by psychoeducation for the child (0.82), relaxation techniques (0.64), psychoeducation for parents (0.45), and relapse prevention strategies (0.45). Notably, techniques in EMDR, such as eye movement/tapping and supportive listening, were excluded early in the coding process because they appeared fewer than three times across all 232 treatments.

While the integration of EMDR techniques for PTSD broadly has been noted (Balbo et al., 2019), controversy on CBT and EMDR’s connections remains. Qualitative studies may be a better way to understand how therapists are using and integrating components of the two therapies (DiGiorgio et al., 2004; Kazdin and Weisz, 1998). Here, we delve deeper into recent findings from surveys using a qualitative inquiry. In particular, Neill et al. (2023) reported substantial integration of techniques across EMDR and CBT in a survey of 346 community therapists (Neill et al., 2023; see also Wampold, 2019) but how this is understood and related to barriers to the use of exposure techniques remains unclear with limited previous qualitative data on this topic. We aimed to present the results of in-depth interviews designed to explore the experiences and attitudes of therapists who treat PTSD in their practices.

In terms of previous relevant qualitative designs, DiGiorgio et al. (2004) interviewed three therapists—one each from psychodynamic, humanistic, and cognitive-behavioral orientations—who used EMDR to explore how they integrated EMDR into their work with clients. Results suggest they all deviated from the standard EMDR protocol to varying levels, with decisions influenced by their theoretical orientation and varying by client needs. These results are consistent with more general practice-focused qualitative studies in this area. In a qualitative study of practicing therapists, Gyani et al. (2015) interviewed therapists to understand their attitudes toward research. They found two different primary themes that they describe as “philosophical issues about the nature of evidence,” and how therapists make decisions (Gyani et al., 2015). Under the “nature of evidence” theme, several therapists felt that RCTs did not contain representative samples (Gyani et al., 2015). Specifically, some therapists stated that due to RCT exclusion criteria, clients with comorbid disorders would not be included in the research trial. However, they are more typical of the clients that they see in community practice (Gyani et al., 2015). Therapists in this study also felt that quantitative research did not provide a full enough picture that qualitative research might, and 18 therapists felt they would rather see studies that incorporated both quantitative and qualitative research (Gyani et al., 2015). All but one of the 33 therapists interviewed felt that the therapeutic alliance was the most important factor in whether or not treatment is successful (Gyani et al., 2015).

Hooyer et al. (2024) examined how therapists in Veterans Administration clinics (10 licensed independent mental health providers) pitched empirically based treatments for PTSD and how they discussed evidence-based psychotherapies (EBPs) with patients. They reported that rich description, patient success stories, inviting language that fit patient needs may foster the selection of EBP’s by potential patients. Their results also suggest that the therapists’ “description of EMDR also illustrates the “unsure” tone when discussing EBPs that often characterized these sessions” (Hooyer et al., 2024, p. 8). These results suggest possible differential attitudes toward certain therapies. However, therapists’ attitudes toward exposure techniques or integration of the various components of different therapies were not specifically reported.

Under the theme of “how therapists make decisions,” therapists interviewed in the Gyani and colleagues study stated that they relied more heavily on their own clinical judgement (Gyani et al., 2015). The perceived rigidity of empirically-supported treatment manuals, as well as clients having a choice in their own treatment, were mentioned by several therapists interviewed as important to their decision-making process (Gyani et al., 2015). The literature suggests that barriers to implementing ESTs include time constraints, excessive paperwork, and lack of reimbursement for activities related to implementing ESTs, such as training and supervision (see Addis et al., 1999). Additionally, clinicians may be hesitant to use EST protocols because of the effect on the therapeutic relationship, unmet client needs, competence and job satisfaction, treatment credibility, hindering clinician innovation, and the feasibility of manualized treatments (Addis et al., 1999).

Dissemination of research and ESTs to community clinicians, which is affected by training, treatment location, and other professional barriers, may play a role in the disconnect between research and practice. This research aims to add in-depth qualitative data about therapists’ use of the components of CBT and EMDR to treat PTSD in their practices. To accomplish this, qualitative interviews were conducted with a sample of practicing therapists. For these interviews, questions were determined and framed around the research question, “what are the experiences of therapists who use components of the empirically supported treatments focusing on Eye Movement Desensitization and Reprocessing (EMDR) and Cognitive Behavioral Therapy (CBT)?” We predicted reluctance in the use of exposure techniques and that certain themes would emerge: (1) themes related to the decision to use aspects of different treatment modalities for PTSD; (2) themes related to how therapists choose the treatment modality for a particular client; and (3) themes related to the use of exposure sessions when treating clients with trauma exposure/PTSD. We expected additional themes from the interviews that might also help understand perceived barriers to the use of exposure techniques and identify how integration of CBT and EMDR techniques may take place in practice.

## Method

### Participants

All participants interviewed indicated that they were trained in both CBT and EMDR in order to be eligible for this study, and this sample of 10 is summarized in Table 1. Nine of the participants interviewed identified their gender as female, and one identified their gender as male. Eight participants identified as white, one as Asian, and one self-described as being of “Eastern European descent.” The interviewees ranged in age from 25 to 63, with a mean age of 42 years. Five of the participants interviewed reported being licensed clinical social workers, or an equivalent name for their state. Two interviewees were licensed professional counselors, one was a licensed psychologist, and two chose “other” and described their license as “LMHC” (licensed mental health counselor) and “LMSW” (licensed master social worker). Nine of the participants interviewed held a master’s degree, and one held a doctoral degree.

**Table 1:** Sample information.

Category	Details
Gender	9 females, 1 male
Race/ethnicity	8 white, 1 Asian, and 1 self-described
Age	25–63 years; mean age of 42 years
License	5 licensed clinical social workers 2 licensed professional counselors 1 licensed psychologist 2 “other”: “LMHC” (licensed mental health counselor) and “LMSW” (licensed master social worker)
Degree	9 master’s degree, 1 doctoral degree
Primary employment setting	7 private practice 2 community clinic or agency 1 “other”—“outpatient VA clinic”

The participants interviewed were located and licensed in Iowa, Louisiana, Ohio, Pennsylvania, Texas, Utah, and Virginia. Four participants were located and licensed in Iowa, and two of the interviews with participants in Iowa were conducted in person, while two were conducted via video conference (Skype or Zoom). All other interviews were conducted via video conference due to the distance.

Four participants had been practicing as a therapist for 5 years or less. Of those four, two had been practicing for 1 year, one had been practicing for 2 years, and one had been practicing for 4 years. Two interviewees had been practicing for 6–10 years, one had been practicing for 11 to 15 years, and three answered that they had been in practice for 35 years or more. All but three interviewees had completed their graduate degree 10 or more years ago. Two of the participants checked that their primary employment setting was a community clinic or agency, seven were in private practice, and one listed their primary employment setting as other and described it as “outpatient VA clinic.”

All 10 interviewees answered that they see clients who have experienced trauma. The participants saw between 16 and 35 clients per week on average for a mean of 25.5 clients per week, with three participants seeing 25 clients per week. Nine of the participants primarily saw adult clients, while one primarily saw adolescent clients.

### Procedures

Participants were recruited as part of a larger mixed-methods study examining therapists’ experiences with delivering empirically supported treatments in online formats. Recruitment followed Institutional Review Board (IRB)-approved procedures and included four strategies: (1) direct outreach by the first author to professional contacts and networks; (2) requests to professional listserv and bulletin board administrators to distribute the study flyer; (3) posts in relevant social media groups (e.g., NASW, APA); and (4) a mailed flyer sent to a random sample of 2,000 marriage and family therapists, due to the unavailability of email addresses. For additional recruitment details, see Neill et al. (2023).

Interested individuals accessed a Qualtrics survey link via email or flyer, completed informed consent, and proceeded to the online questionnaire. At the end of the survey, participants could provide their email address if they were interested in participating in a follow-up interview. Those who opted in comprised the sample for the current study. Inclusion criteria required participants to be licensed mental health professionals (e.g., social workers, psychologists, marriage and family therapists, and mental health counselors) who spent at least 50% of their professional time providing individual therapy.

#### Interviews

Interviews were approximately 1-h long and conducted with practicing community therapists. Participants were asked to set up a mutually agreeable time for the investigator to come to their office, meet at a mutually agreeable location like a coffee shop, or come to the investigator’s office at a midwestern university’s campus to participate in the interview. For those participants not in the Central Iowa region, a remote video or phone interview option was offered. With the permission of the participant, the interviews were recorded (audio only) so that they could later be transcribed and analyzed. The interview questions, listed in Table 2, included items such as: “What types of problems/disorders/diagnoses do you use CBT/EMDR for?” and “Are there any problems/disorders for which you feel using CBT/EMDR is inappropriate?”; however, the interviewer did *not* ask for any identifying characteristics of clients. The University Office for Responsible Research’s Institutional Review Board approved this study. De-identified data will be made available upon reasonable request.

**Table 2:** Interview questions.

Question
What made you decide to get trained in CBT/EMDR? Why do you use it in your practice?
Where did you do your training?
Did you have to complete supervised hours for your training?
Do you have any type of certification that you are trained in CBT/EMDR?
What types of problems/disorders/diagnoses do you use CBT/EMDR for?
Are there any problems/disorders for which you feel using CBT/EMDR is inappropriate?
On average, how many sessions of CBT/EMDR does your “typical” client need?
What do you think is the “mechanism of change,” or reason, CBT/EMDR works? (What part of it?) You noted that you do not use (ex: exposure) for CBT/EMDR. Can you tell me more about that? Why do you not use (ex: exposure) when using CBT/EMDR?
Does this ever change by the type of problem your client has?
I am interested in studying the way clinicians, such as yourself, use treatment manuals in (CBT/EMDR). Is there a treatment manual you use for (CBT/EMDR)? What is it? Do you use all of the manual? What parts do you use and why? What would make you decide to use all of a manual (follow a complete manual)? What could researchers do to improve treatment manuals for clinicians?
Open-Ended Exploration Questions:
What ideas do you have for ways that researchers and clinicians could work together for the benefit of clients? Is there anything else you think it is important for me to know?

The CBT/EMDR questions were asked in separate sets relating specifically to CBT or EMDR (e.g., first let us talk about CBT when those were completed, then let us talk about EMDR).

#### Qualitative data analysis

All interviews for this study were conducted by the first author, secured by encryption and transcribed by the transcription service Rev.com. After receiving the interview transcripts from Rev.com, the first author (EN) read over the transcript while listening to the audio to ensure accuracy and correct any mistakes. Themes from the interviews were discerned both within and between participants. The transcriptions were verbatim of the interviewer’s question and participant’s responses. The interviewer also took detailed notes. These include any important behavioral or gestural acts not apparent in the verbal transcription (e.g., display of affect and body posture).

Interview questions were framed broadly around the research question: “What are the experiences of therapists who use components of the empirically supported treatments Eye Movement Desensitization and Reprocessing (EMDR) and Cognitive Behavioral Therapy (CBT)?” Follow-up questions were inspired by participant responses to questionnaires completed prior to the interview (described in a previous study, see Neill et al., 2023). For example, a participant who indicated they used CBT to treat clients who have experienced a traumatic event, but did not use exposure sessions, would be asked why they omitted exposure, with the goal of better understanding how they make decisions about empirically supported therapeutic components for their clients. Interviews were framed so as to avoid the appearance of bias on behalf of the interviewer by using the set of questions in Table 2 as a semi-structured interview guide with open-ended, neutral delivery of questions to ensure consistency and reduce leading or loaded questions (Noble and Smith, 2015).

Qualitative data were analyzed using an inductive coding approach, guided by grounded theory (Field, 2013). Grounded theory is the “discovery of theory from data,” which can be “systematically obtained and analyzed in social research” (Glaser and Strauss, 1967, p. 1). A complimentary, systematic methodological approach to understanding qualitative data described by Willms and colleagues (1990) was also used. Beginning with interviews and field notes taken during interviews, “Coding Consensus, Co-occurrence, and Comparison” was used to identify themes in the interview data (Willms et al., 1990). Looking at the data from all the interviews conducted, patterns and similar statements across participants interviewed were identified, especially those issues and themes that seem to apply to all or more than 50% of participants. A list of words or phrases gleaned from the interviews and field notes was identified, which includes themes, issues, actions, cultural determinants, and symbols relevant to participants’ experiences with EMDR and CBT. Each of these “codes” found in and across interviews was defined and organized into larger themes. Specific quotes are used to help illustrate each theme. This type of qualitative coding has been used in previous qualitative psychotherapy research (Palinkas, 2014; Reding et al., 2016; Willms et al., 1990). This methodology allows for the accommodation of both *a priori* and additional themes in qualitative data. Willms and colleagues (1990) argued that this methodology, or systematic approach for using qualitative data and analysis in research, is an essential precursor to culturally effective interventions in clinical and community settings. Therefore, this is an excellent fit for the type of data gathered in this study.

## Results

The themes identified were consistent with *a priori* expectations and were categorized in the following manner: (1) what kind of treatment modalities were utilized for PTSD/how participants choose techniques for each client and (2) the use of exposure sessions when treating clients with trauma exposure/PTSD. Several additional themes were identified and labeled as follows: single-incident versus complex trauma, EMDR versus exposure sessions/therapy, EMDR versus CBT, financial commitment in EMDR, and perceptions that EMDR “sounded crazy.” In the following, each of these themes is described with illustrative text of responses.

### *A priori* themes

#### Type of treatment modalities utilized and choosing techniques for each client

As noted, all participants interviewed indicated that they are trained in both CBT and EMDR in order to be eligible for this study. Each interview participant was asked about the other types of treatment modalities they use with clients to better understand their clinical practices. Most participants who were interviewed responded that they use multiple treatment modalities, with a few describing themselves as “integrated.” One said, “Everything is integrative you cannot just use… No. I do not just use one modality.” While some participants interviewed were loyal to using EMDR mainly, most stated that they still have other modalities that they use. “I mostly use EMDR, but I weave in some of the CBT tools,” one participant shared. Another said, “I use a lot of interpersonal stuff. I think that’s probably one of my favorites. I use narratives sometimes. I’ve used trauma focused CBT, but not as much. I use ego state. Solution-focus.” A few participants mentioned other modalities that focus on sensations in the body. One stated, “Apart from the CBT and EMDR, I use a lot of body centered approach it’s like a sensory motor.”

One participant said:

I am truly that integrated therapist. I can speak different languages and understand which ones we are doing. I used to sit with a CBT therapist and she and I would talk. We were like, “We’re doing the same thing.” She goes, “Yes, we are using different language.” … We would talk about what’s the most important part of therapy and what makes it work: relationships and trust. You have to be willing to sit with them. Then of course there are specific tools you use to get there.

One participant’s quote illustrates why many therapists describe themselves as “eclectic” or “integrative.”

I think for me personally, EMDR was definitely a better fit, but I have seen success with TF-CBT. I have seen success with cognitive processing therapy. But I am much less likely now to say that I am eclectic than probably I was when I got out of grad school. I’m much more likely to say different things work for different people. And so, my responsibility is to help you figure out what I can offer that might work best for you or to help you find someone that might be able to offer something that works better. But I do not think any one intervention fits for everybody.

Another participant, who also considered themselves a specialist in EMDR, said something very similar during their interview.

You’re going to do different things depending on the issue that presents and what someone wants to do… The most important thing is the relationship… Everybody knows it has to be a good fit. Then we can decide which treatment we want to use.

Most participants agreed that choosing a treatment modality would depend on their client–client characteristics, as well as their goals for therapy. One participant said:

Well it totally depends on the client… it depends on their goals. I do a lot of trauma work and so a lot of times, well, I do not know – like framing negative self-statements for example – I might have that as a goal, we might agree on that as a goal, to you know increase their level of positive self-statements… With just clients that I saw yesterday, I saw six people, six women, who were in domestic abuse situations yesterday and then three with other concerns. And so, changing their view of… increasing their self-efficacy, for example, would be a goal. Decreasing their self-criticism… increasing their assertiveness, and then we might move into other things like, well, this would not really be CBT but behavioral, improving their sleep hygiene, improving their medication compliance, and strengthening their ability to disconnect from their husband and conflictual situations. So all those things would be woven in depending on the circumstance.

How do therapists decide what treatment modality or technique to use? Most participants acknowledged dependency on patient goals and characteristics such as readiness to confront trauma, adaptive coping skills, and depth of support systems. However, some participants were loyal to a single treatment modality. These interviews showed that some participants make the decision about what modality to use for a certain client primarily between CBT and EMDR.

It depends on how much they believe the past is still affecting them. Like if they know this junk is still haunting me and I tell them about EMDR, they are like, “Yeah! Let us do this!” But the people who do not… If I feel like they are not even going to be willing, I probably do not even approach it and I just start using CBT.

Another participant stated they use CBT for:

Your basic depression, anxiety, and… things like that, as well as with complex trauma. I use CBT kind of as a jumping off point before I start EMDR. I use it in conjunction with DBT and a couple of other things, kind of to give clients the skills that they need to be able to make it through EMDR.

Another stated, “CBT you know I – you intermingle that kind of often with all clients unless they just need processing support. For EMDR I have some clients that are specifically seeking EMDR. And then if they are ready you can work them through that.” But how do therapists know when a client is ready for EMDR? One participant put it well when they said:

I always want to know how much emotional regulation skills they have first. Because if I feel like they cannot even manage a bad day, then working through a bunch of crap is not going to be so great. Though, that’s when I do the skill building, but if they tell me, “I’ve been in therapy before, these were the coping skills I know when I’m feeling crappy,” whatever, then we can just jump in right away cause all that other works done.

Overall, the participants in this study who were trained in EMDR want to use that modality with their clients who present with symptoms of PTSD or who have a trauma history. However, they cannot use that modality if the client does not have some coping skills to rely on while processing the trauma. CBT often serves as a starting point for many participants before transitioning to EMDR, particularly when working with clients who have limited resources. One participant stated that clients were a good candidate for EMDR if they were “stable, they are not dissociating… substance abuse is not out of control, like they are not full blown in addiction.”

#### Using exposure sessions for trauma exposure/PTSD

Nine of the participants discussed their use of exposure techniques in therapy, and each stated that they did not use exposure sessions in their practice for trauma exposure/PTSD. Their reasons varied, but one participant summed up these findings well saying, “EMDR works a lot better than that.” Another participant discussed a similar opinion saying that they do not use exposure sessions or CBT for trauma and PTSD anymore because they found that EMDR was just better for the complexities of the cases they were seeing. This participant discussed the intensity of the trauma their clients had and the fact that the trauma was not from a single event but a multitude of events. They found that sexual trauma, particularly childhood sexual trauma, was especially difficult. Another stated:

No. Certainly if I work with somebody who has a phobia, even with EMDR with a phobia, there’s an aspect of now you need to go do the phobic thing or see the phobic object. So there’s that aspect of exposure, but not exposure therapy.

Another participant’s response had a similar theme:

Most of the clients I’m seeing have that complex trauma, like I said, where there’s past abuse or neglect or abandonment, things like that. And so… yeah, unless I’m going to force them to talk to their parent who they are not allowed to actually have contact with…

Another participant felt similarly about the clients they saw, saying:

It’s just not a tool that’s appropriate. So nobody comes in for exposure. How do I learn to adapt to, ‘My husband’s beating me?’ How do I learn to… I mean I do use the EMDR flash technique, for intrusive memories of incest that are too distressing so we’ll do the tappers and the prep and the flash and the rating of numbers, but I weave that into what’s going on at that moment when that person had a dream about a certain situation that’s triggered by them, not by me.

One participant gave a better picture of how they feel that EMDR works better than CBT-based exposure sessions for the treatment of trauma exposure and PTSD. “No, I actually would say I personally do not do exposure therapy. I do not walk with them to the airport. Instead, I use people using their mind, walking through the movie, but that’s when I pull in EMDR.”

One participant felt strongly about not using exposure sessions for trauma survivors in their practice. When asked about using exposure sessions, they stated, “BIG OLD NO, EMPHATICALLY NO!” When asked why not, they stated:

I tell all my clients, “Do not feel obligated to share a painful thing,” and to not think it’s necessary to in order to heal or feel better whatever you call it. And that’s just because for a lot of the clients here, particularly my caseload, it’s not just your one or two traumas it’s, it could be a lifetime of traumas. And so I’ve seen a lot of people even just maybe starting to share a story because that’s what they think there is – almost just immediately flood and just become overwhelmed emotionally. So, I feel that by me emphasizing you do not have to share painful stuff, unless you want to that gets more buy in of sorts as well as the people who perhaps I do not emphasize that too I’ve noticed some uh their lack of follow thru or just not coming back. And I’ve had quite a few clients who have done some – what sounded like some exposure work – and while they have noticed perhaps some relief in certain aspects, in that moment when they were doing it, it was not fun at all and that emotional memory of therapy not being fun is another pretty big barrier to people coming in to seek services or maintaining sessions.

Participants said they saw clients who had many barriers to coming or continuing to attend treatment for trauma. Anything that may further impede them from coming into therapy is not desirable, and the participants try to avoid exposure sessions so that clients return for further services and get the help they need. Another participant mentioned a similar topic, saying they do not use exposure sessions because:

Sometimes I do not get that far. Their follow-through is not good enough to get to that place. The cognitive processing therapy folks that have intentionally signed up for that piece have been a little more amenable to it. But often it’s been that they do not stick around that long. They get to feeling a little bit better and they take off before we get to that part… I have trouble sometimes getting a narrative that it seems would actually be helpful to them, not a three sentence, “I went to Vietnam. It was horrible. I came home and it was horrible.” Getting a longer, more meaningful narrative.

Some participants offered other alternatives to using exposure sessions with CBT and EMDR. One said that they did not use exposure sessions, and that “usually when they are, clients that have trauma that’s significant enough that it’s impacting their daily life and I’m not using EMDR for whatever reason… I tend to also to use internal family systems more than CBT.” Another differentiated between using “prolonged exposure” and what they termed “cognitive exposure,”

Not prolonged exposure. I do a little bit of cognitive exposure with a trauma narrative. And doing that, if they choose to do that or the amount of exposure that they get in EMDR depending on what they are working on, but not an intentional exposure.

Being asked about a component of CBT led the participants interviewed to directly compare exposure sessions of CBT to EMDR. In particular, some of the descriptions of how EMDR worked “better than” exposure sessions sounded more like imaginal exposure. Some participants were asked more about this. One participant thought that EMDR was the same as imaginal exposure, “…you have imaginal exposure, you have *in vivo* exposure, so EMDR is all imaginal exposure, which to me is just as profound as being there actually, in fact, I’ve noticed that our imaginations are way more real than reality sometimes.” When asked to expand more about what EMDR may be like in exposure sessions, their answer included information about the graded exposure hierarchy:

It’s more targeting memories so you know you find your touchstone, or you know the “worst” are the first, whatever, all the other memories that are kind of in the same vein, yada, yada, yada and so we start with the touchstone or the “worst” from there and I let the client decide which one they want to do next. So, in a way that’s kind of a hierarchy and in a way it’s kind of not.

Another participant also felt that EMDR was an “exposure therapy.” They said, “Well, you cannot do EMDR without having exposure sessions, and TF-CBT has the trauma narrative, which is considered exposure, as well. I build up to that. That’s not session number one. But yes, eventually in the process, that’s the goal.” This participant wanted to use exposure sessions when using CBT, thought of EMDR as exposure-based therapy, and had a goal of using various modalities with their clients. The same participant, however, stated that they did not need the person to give all of the details of the traumatic event in order for EMDR to work.

I do not need to know it for it to work. The exposure has nothing to do with whether or not I know it. Whether or not they say it to me, whether or not they tell it to me, I do not need all the nitty gritty details for EMDR to work… The client knows what it is. It does not matter if the therapist knows what it is. I do not need to know the details. The client’s getting exposed to it inwardly, in their thoughts and in their experience of the memory. Whether or not they are saying all the nitty gritty details to me is irrelevant. What’s crucial, though, is that the client is actually going there. You know. If they are saying it, but you know, if they are saying, “Yeah I see it in my mind, yeah I’m thinking about it,” they are giving me things that they are observing, that kind of thing, but they are not actually going there, well then, we are not doing exposure. It’s sort of about teaching the client how to do the work.

This participant went on to directly compare EMDR and CBT with exposure sessions:

What I like about EMDR’s approach over TFCBT, is that I think it is less intrusive. The client is in the driver’s seat, so they get to set the pace. They can press pause whatever they need to, and they have the ability to decide how quickly they want to go through it. With the trauma narrative, it does not give the client the ability to set the pace as easily. And in the TFCBT protocol, there’s oftentimes a sharing of the trauma narrative with a loved one or a trusted individual. And that sometimes can be really intrusive. So, there’s that part. And the nitty gritty details of every aspect of the trauma, that’s not always available. So, a trauma narrative, especially something that happened if the trauma’s from pre-verbal, you are not going to be able to write about it, because you do not have cognitive memory of it because you were not cognitive then. It’s incomplete, I think, and it’s intrusive. I prefer the EMDR approach, because it leaves the client in the driver’s seat and it allows you to work with the parts of the memory that are necessary to reprocess, without going into more than is really necessary. And you do not have to have the whole story in order to be effective.

A different participant had given a lot of thought to whether or not EMDR was a type of exposure therapy:

Well, I’ve thought about whether it’s an exposure therapy, and I can kind of see why people would say it is, because you are doing *in vivo* exposures if you will, or imaginal ones, so they are imagining it while you are in session. I can see how it can be seen as an exposure for anxiety. Because with anxiety or even OCD, you can do future templates where you can think about what’s the worst-case scenario and then they can imagine that scenario and how they work through it mentally and work through all the emotions. That’s how they can, and it actually can impact them in a positive way. So, I can see why people would say, because that is an exposure, that is an example of an exposure.

But I went to a training recently that really helped me clarify this more, especially that thought of EMDR is just another subset of CBT, it’s just an exposure therapy basically, or you know, kind of a reduced exposure therapy, not going into it too much, you are not flooding them, but it is exposing them. But I really loved the training because it really, actually it was Francine Shapiro talking about EMDR as an actual therapeutic model. Not just a protocol. Not just a CBT protocol, but an actual therapeutic framework and how do we view EMDR like we view psychodynamic theory? Or all these other theories that we learn about in grad school, how can we use EMDR as a framework for the work, not just as this is just another protocol that’s falling under CBT. So since then I compare it, like there’s a lot of overlap to EMDR but it’s because I think what I would say to somebody who’s arguing it’s just an exposure therapy, I would say yes, I would see why you see that because there is kind of some overlap, but EMDR is not encapsulated in only that future template, so that’s one aspect of it. But we are doing EMDR in our framework the entire time from the moment the client comes in because our perspective is really on seeing diagnosis even, it’s seeing the experience that clients are going through as their unprocessed memories that they have in their minds. And because of these unprocessed memories, it’s impacting their actions and thoughts in the way that clients are in the present. And so if we only just limit EMDR to just manual in the sense of it’s just that, then I think we are really underestimating what EMDR is. If you just stress the eye movements or whatever.

One reason many participants gave that EMDR and imaginal exposure techniques were not the same was that in EMDR the client only has to give the participant a picture or name of the event. They do not have to go into details about the traumatic event, as they would need to do with *in vivo* or a CBT-based exposure session. Here are some ways that the participants elaborated on this idea.

Well to target [the memory] they have to just give me a brief bullet point of what that memory is. But once they are working through it in their own mind, I do not really need to know what’s actually happening with the memory, I just want to know what’s changing with the picture… What’s going on in their body with their emotions. So they do not have to tell me the whole memory – they do not even have to tell me most of the memory just a smidge and the rest is up to them.

One participant was more adamant that EMDR is not an exposure-based therapy for this reason—that the client can give the name of the picture, and in fact is asked not to go further into the traumatic event.

So, first of all, when you are doing your history, you are taking in your assessment, you know I was trained, you know you are very careful not to have people describe the trauma then. You frame it like, “You can give me a cue word or you know one sentence…” We’re not going to, we are just going to title it, we are not going to go into that trauma now. So, you are very, of course things could be triggering to clients when you are assessing them, but you are very careful, I’m very careful not to re-traumatize them during the assessment phase. And then, again how I was trained and what I was told is it’s not exposure therapy, because that’s the whole point of the bilateral stimulation is you are keeping them grounded in the present, so that they are fully online in the present. I mean and sometimes it’s hard to tell so you have got to really pay attention. So, they have got one foot here in the present safely here and then one foot back in the trauma and so it’s not like exposure therapy, they are not going fully back in. They’re kind of dipping in and then coming back, so it’s very intentionally not exposure therapy, in my opinion, and training.

Another participant described this as asking clients for a “book title” about their traumatic event:

I tell people that for the events on the target sequencing plan to just perhaps think of giving me the, kind of saying it like the title of a book, you know – “I Got Punched,” or whatever. And then when we target something, I tend to ask people to just let me know perhaps the part that represents that moment the most or the worst part.

When asked whether or not their clients, with whom they were using EMDR for trauma, did end up telling them more than just a name or picture, many responded that their clients do eventually tell them more about the traumatic event. One said:

Most of them do… I think it’s in a way comforting for them to know that someone else knows what happened. And I’ve also noticed that as they process they say, “What’s different now?” They’ll be really descriptive about everything that happened each time. They do not have to be, but they choose to. So, in a way I’m hearing the whole memory with that as well. But if all I know is, “I was date raped,” Cool – that’s all I needed to know. It’s easier on me actually.

Another participant spoke more about how they prepare clients so that they know they do not have to talk extensively about their trauma. Some participants feel that this should be emphasized more for certain clients:

It’s totally up to the client, it’s very individual. And so I say to them, you can say as much, you know… The directions say, you know the script or something and I use a lot on script because I like it, the one that I was trained on. I think it says you know between sets, “Please tell me as clearly as possible what is happening for you?” But a lot of that is what are you noticing in your body, what feeling is coming up for you? And so I think for people that are like super traumatized and very avoidant about talking about the subject material, I think I take extra care to say, “You do not have to tell me what you are seeing, you know, I just need to be checking in with you about… You know you can say, ‘I’m seeing the same thing or the image has shifted.’” Or so if people that are really avoidant about it or have a lot of shame about talking about it, I’m even more clear with them, “You do not have to verbalize exactly what’s happening in that picture.”

One hypothesis about this idea is that in EMDR, you do not have to talk about the memory itself, but many clients do, so that if you are coming to therapy, you probably want to talk about the event or at least expect to have to talk about it.

### Additional themes identified

#### Single incident vs. complex trauma

Another theme found in this interview data was participants commenting on trauma itself. That is, several different participants independently, and without a question prompt from the interviewer, talked about how prevalent and common trauma is in their clients’ lives. One said, “I think that life has a lot of trauma exposure in it, generally. So, if we talk about big T trauma and little t trauma, everybody has trauma.” [To clarify, “Big T” (Trauma) and “little t” (trauma) trauma are terms that Francine Shapiro used in her initial trainings for EMDR (Shapiro, 2001)]. Perhaps, the participants have more of a trauma lens, and therefore do see, or at least believe that they see, trauma in their clients more frequently. One said, “I see everything, but I find that a lot of things are rooted in trauma because that’s the lens I see things.” Another participant stated trauma exposure is pervasive on their caseload as well, “I would venture a guess that at least 90% of my caseload has been through traumatic events. And I think the stats, or I think roughly about 66 or 70% of people in outpatient treatments have experienced trauma as well.” This participant believes that even therapists who do not specialize in trauma treatment see an extensive amount of trauma exposure in their clients. Another participant stated, “I’m in private practice, so I take pretty much whoever comes my way. But it’s pretty rare that I encounter someone who does not have some sort of trauma, whether it’s full-blown PTSD… There’s oftentimes some traumatic experience there.” This participant clarified in EMDR terms as well, saying, “I see lots of people with ‘small “t”’ traumas, not full-on PTSD, but a lot of ‘small “t”’ traumas, because I think we all do, personally.”

Furthermore, many participants discussed trauma in terms of single incident trauma compared to complex trauma, which became its own theme in the data. This can be seen in many of the other themes and questions explored in the qualitative data above. In fact, seven of the ten participants interviewed directly addressed this topic, even though it was not directly related to an interview question. Most of the participants, especially those who had been in practice for a few years, had seen a client who had a single incident trauma, but these clients were very much the exception in their practice. One participant said, “Most people have piles of different trauma. I have one teenager that had one trauma, and it was, they are easy to clear up and make progress. Most people are complex and harder.” Another participant had a similar story about a case with a single incident trauma, who was a rape survivor. They said, “That’s unusual in my practice. That’s not a typical client for me. I have more long-term cases than short-term cases… I’d love to have some of those short ones. It was really fun. But I do not get a lot of that.” Some participants felt that clients with complex trauma were not only the norm for their practice, but those clients were, in some ways, used to trauma in their lives. This was an important consideration in their treatment.

I tell all my clients do not feel obligated to share a painful thing and to not think it’s necessary to in order to heal or feel better whatever you call it. And that just because for a lot of the clients here, particularly my caseload, it’s not just your one or two traumas, it’s, it could be a lifetime of traumas… A lot of my clients tend to cope well enough as is and them kind of having lived through many traumatic events they, I mean, that’s kind of their threshold of normal.

Some participants who thought of clients with single-incident trauma thought that those clients would not necessarily qualify for a PTSD diagnosis, but still experienced an upsetting traumatic event in their lives that they wanted to process.

With one [person]… They had no history of trauma, but they were in an abusive relationship and they were able to resolve that within six sessions… I would not say that person had PTSD per se, but a lot of trauma reactions. But for anybody in my experiences who’s gone through extensive sexual violence, sexual trauma, especially childhood sexual trauma, or if they have been adopted, gone through foster care, or if they have been in a lot of ongoing traumatic events, situations throughout their whole lives, potentially then to the point where they have dissociation going on, those situations, I feel like it’s hard to put a timeline but it’s definitely more than just a few months. I’m guessing probably they are going to be in therapy for longer than a year, for sure, to work through everything.

Complex trauma is so prevalent that one participant stated they could not think of any clients who had a single incident trauma. “I’m thinking, if there’s any that do not [have complex trauma] … and if there are, they are not coming to mind.” Another participant also could not think of any examples of a single-incident trauma.

Honestly, in all my years of practice, I do not think I’ve ever seen anybody that just had one trauma. They do not end up in therapy because they saw one thing. Unless maybe they are in some sort of a structure, like in a police situation where they are doing debriefing, but they do not end up seeing a psychologist in individual psychotherapy. As a result, yes, all of my clients have had multiple traumas, frequently multiple extreme traumas.

#### Barriers to EST

One of the goals of this study was to better understand what was actually happening in therapists’ offices. In the United States, healthcare and insurance policies result in access and treatment option disparities. Therefore, the types of people who can access a therapist in private practice may be very different from those who access mental healthcare through a community mental health provider. The current study was able to interview therapists in a variety of settings to better understand their mindset, practice patterns, clinical reasoning, evidence integration, and the types of clients they are seeing. One participant helped to identify a particular barrier to treatment, especially in community mental health, where clients are likely to have less resources—both personal (coping skills, etc.) and financial (health insurance, transportation, etc.).

I work in community mental health, the agency that I work for is the Medicaid provider for my area. And so, we have a lot of clients who start but never finish treatment. And so, a lot of them ever only get CBT, because that’s as far as they get. Because before I start EMDR, I want to make sure that they can actually… they will actually follow through with treatment, because I do not want to start something, and dredge up memories, and you know, all of that, without knowing that they are going to come back next week.

Another theme or barrier in community practice is how often or how well clients are completing homework from CBT sessions. One participant said:

My luck with homework has not been super. With my cognitive processing folks have done well with it. My others, it’s kind of hit and miss. But I usually give them some try this at home kind of piece. “Stop and notice when you are thinking this way, and can you jot a note. What else is going on? Stop and notice when you are thinking this way…” I would say I probably rarely get someone to bring that back. They’ll talk sometimes about having done it, but I do not have a lot of success at getting them to bring those back.

#### EMDR sounds “crazy”

The idea that EMDR sounds “crazy” or like it should not work, became a theme of its own, as several other participants mentioned this initial feeling or reaction in themselves or in their clients. One participant stated they “thought it sounded like voodoo or magic” when they first heard about EMDR. This participant also looked at the research, both the research comparing EMDR to CBT, which they said “people consider the ‘gold standard’” in the trauma field, and the research on the protocol of EMDR itself, and felt that they understood why EMDR really worked. Another participant shared something similar, stating, “I had a young colleague… who said, ‘Let us go get trained in EMDR.’ I said, ‘No. That’s BS. I do not need to be a monkey and wave my fingers around.’ She said, ‘No, I think that EMDR really is something that is evidence based.’” Participants reported that they thought the idea of EMDR sounded crazy or like it would not work, but suggested that research they read or colleagues who were trained began to change their minds. At the time of their interviews, all ten of the participants interviewed felt very positive about EMDR, especially as an effective treatment modality for their clients with PTSD or who have experienced traumatic events. However, they still recognize that many people will find the concept of EMDR a bit strange.

Many participants also commented on what their clients think about EMDR. While some clients are looking for EMDR therapists, others are introduced to the idea of treatment modality by their therapist. They also noted some clients are willing to try anything for relief of their symptoms, but many remain at least somewhat skeptical of EMDR. One participant stated that their clients “think I’m kind of crazy… Most of them, their eyes get a little bit big when I talk about [EMDR]”. Some participants say that their clients come to them specifically for EMDR, or that clients get to them after trying other therapies that have not worked, and they are willing to try anything for some relief of their PTSD symptoms. One participant said, “Every once in a while, I’ll encounter someone who’s like, ‘Well that’s really strange.’ But usually, if someone is coming to me who’s not specifically seeking EMDR, I spend a lot of time educating them about it and why it works.” One participant also thought their clients would “think it’s [EMDR is] creepy or weird, but most people are really open actually. I’ve surprisingly found that more people than not are really receptive to the idea.” Finally, one participant noted that a shift in the field of trauma and mental healthcare has helped buy-in for EMDR treatment for PTSD and trauma. “I think it’s becoming increasingly popular. The VA likes EMDR, so that’s been helpful in getting people’s buy-in, and certain insurance companies are endorsing it a little bit more. So that makes a difference.” Another participant interview summed this trend up well, saying:

We now have a lot more people who are actively interested [in being trained in EMDR]. I also find that EMDR is being talked about a lot more now. So EMDR itself has grown. [There are] about 8,000 [members] in the EMDR Association which is up from… it’s probably doubled in the years that I’ve been involved. So, it’s significantly bigger nationally. And I think on a local [or] state level it’s bigger. So yeah, I think there are a lot more people.

#### Financial commitment in training

One participant had an interesting perspective on *why* therapists may be “misusing” treatments or trying to get one treatment to many different presenting problems or disorders. They started by saying, “All the trainings are expensive, and non-profits are – you have got to wait until the right grant comes along.” This participant worked at an agency that actively sought grants to fund therapist training. However, therapists were still trying to piece together therapies for their clients. This participant stated:

I really like training. I’ll get trained in anything there is, but in a community setting, you do not have funds for that. There’s not funds to do “CBT for Depression,” and “CBT for PTSD,” and “CBT for Insomnia.” You take the CBT that you got in grad school, and you pull out your basic textbook. And that’s what you do because that’s your training in CBT. And then you tweak it for whatever it is. And so, it’s an interesting thing to be in a place that they say, “Okay, here are the things that CBT works for,” and they have got a specialized target for that. And then they actually provide consultation to make sure that you are keeping some model fidelity.

Having EMDR therapy done as a client is expensive as well, as one participant interviewed acknowledged.

And it’s expensive. If you want to go and see an EMDR therapist, like if you or I wanted to go and see an EMDR therapist, the chances of finding one that takes your insurance is slim. And then, if you do not… I know one of the therapists in my office who was previously EMDR trained sees people as a private practice, charges, I want to say… it’s not quite twice as much for EMDR as it is for regular talk therapy, but it’s close, it’s a lot more for the EMDR than it is for the individual therapy, for like the regular talk therapy.

Not only is it expensive for therapists to be trained, it is also expensive for clients who want to access EMDR treatment. Therefore, this means that some individuals are not able to access this empirically supported treatment.

#### Using EMDR themselves

A final potential theme worth noting is that three participants mentioned having EMDR done on themselves before being trained in it. Two of these participants found EMDR to be so effective that they wanted to be trained in it themselves in order to better serve their clients who are trauma survivors. One of the participants actually had a bad experience being treated with EMDR, and thought, “EMDR is a bunch of hooey! I do not want to touch that with a 10-foot pole!” However, as they read more about the treatment and learned more about it, they realized that the therapist who had treated them had not been using the treatment correctly. They stated after looking into the research and taking the first part of EMDR training that they “decided, this is really something I believe in, and so I kind of drank the Kool-Aid, and now I’m really into it.”

## Discussion

Results of this study add to the evidence that community therapists are integrating techniques across treatment modalities (Neill et al., 2023; Wampold, 2019) and help clarify how therapists understand the use of these techniques and the barriers to their implementation in PTSD treatment. More than 20 years ago, Addis and Krasnow (2000) suggested that more information was still needed about how clinicians were using ESTs and treatment components in clinical practice, suggesting that there was a great need for qualitative data asking therapists directly about this topic. Gyani et al. (2015) also suggested that qualitative studies may be one way to bridge the gap between research and practice, 15 years later, by gathering more information directly from practitioners in the field. This research was certainly correct, and our results suggest that such information is just as important today. The themes identified in our results provide insight into what kind of treatment modalities (and what parts of those modalities) therapists are using for trauma and PTSD, as well as how therapists are choosing what they feel is the best set of treatment techniques or modalities for each client.

The results of this study illustrate how practicing therapists think about the treatment modalities broadly and their thinking around the specific components commonly found in effective interventions. As noted, Chorpita and Daleiden’s (2009) application of the distillation and matching model identified core elements—such as exposure, cognitive strategies, psychoeducation, and relaxation—as the most frequently used in successful RCTs for child trauma. This approach shifts the focus from rigid adherence to manualized treatments to a more flexible, evidence-informed strategy that emphasizes the most effective ingredients. Earlier work by Chorpita et al. (2005) supported this by proposing a model that matches these distilled components to client needs based on presenting problems or demographics. This component-based framework not only helps clinicians tailor interventions more precisely but also addresses gaps in the literature where full protocols may not be feasible or well-studied, such as with EMDR in children or the limited role of supportive listening in trauma-focused care.

While the integration of EMDR into psychotherapy for PTSD broadly has been noted (Balbo et al., 2019), our data suggested general acceptance of EMDR and that it may be a way that therapists overcome barriers to the use of *in vivo* exposure techniques in practice settings. Interestingly, none of the participants in this study were using exposure sessions when using CBT. In fact, these participants only use CBT elements in the beginning of treatment for trauma survivors, and then usually move onto using EMDR to treat trauma exposure. However, with further probing, several of the participants actually considered EMDR a type of imaginal exposure. As noted, Hooyer et al. (2024) examined how therapists discussed treatments for PTSD and reported that “…EMDR was presented as effective but without a “confident” tone toward its efficacy” (p. 8). The theme that “EMDR sounds crazy” was consistent with their finding that therapists “like” the therapy, though it may suffer from a marketing issue. Our findings suggest that one mechanism of action may be EMDR’s ability to implement exposure techniques in a more user-friendly manner (with therapists as the users). A potential avenue for future research—before firm conclusions are drawn.

Many interviewees seemed to understand “exposure techniques” as only *in vivo* exposure. Interestingly, many EMDR participants thought that EMDR sessions were, in fact, imaginal exposure. Even though clients do not have to give details of their experience (they can tell the therapist only the name of a “picture”), most participants stated that most clients do in fact tell them more about the traumatic experience, and the client should be moving through these pictures and experience in their mind. This really describes (imaginal) exposure techniques, thereby showing that perhaps EMDR and exposure sessions in CBT are actually not that different from each other. However, future research or training programs could perhaps better define imaginal and *in vivo* exposure so that participants and researchers can be sure that they are discussing the same concepts. As noted, Neill et al. (2023) surveyed 346 therapists in practice settings (78.61%) were trained primarily in CBT and 135 participants (39.02%) were trained in primarily in EMDR and found that psychoeducation about trauma was the most common element used. These findings also suggest underutilization of *in vivo* exposure techniques across therapists.

Over 20 years ago, Addis and Krasnow (2000) suggested that better communication and collaboration are needed between researchers and clinicians about how the components of EST manuals are working in practice. That is, how are EST components working outside of a controlled, clinical environment? By speaking in more depth with each participant, we were able to better understand how ESTs such as CBT and EMDR are implemented in practice and how therapists feel their clients are generally responding to each treatment. Another finding supported by previous research was that many practicing therapists feel that RCTs do not contain representative samples and that due to RCT exclusion criteria, clients with comorbid disorders would not be included in the research trial, but are more typical of the clients that they see in community practice (Gyani et al., 2015). Participants in the current study agreed with this and felt that researchers needed to do a better job of researching the types of clients that they actually see in practice. Efforts may be needed to identify clinician-friendly predictors of favorable outcomes for tailoring the selection of elements (Weems, 2019). Having packages of components may have downstream prevention effects by targeting core symptoms (Weems et al., 2021) that may be more or less salient for a particular client. Our results suggest this may help frame the implementation of ESTs for clinicians and also help them pitch ESTs to their clients (Hooyer et al., 2024).

Additionally, previous research showed that therapists felt the therapeutic alliance was the most important factor in whether or not treatment was successful (Gyani et al., 2015). The perceived rigidity of empirically-supported treatment manuals, as well as clients having a choice in their own treatment, were mentioned in previous research as important to therapists’ decision-making process on how to treat a client (Gyani et al., 2015). The current study also found that many therapists mentioned they make decisions based on client need, and different EST components were endorsed regardless of which type of treatment or treatment manual they were a part of. Replication of these types of qualitative findings is an important contribution to the field, as the small sample sizes sometimes call into question the generalizability of the findings. However, this study found similar themes in our participants’ interviews and replicates many of the same ideas brought up in the Gyani and colleagues’ study.

Despite the strengths of this study, there are also several limitations. Therapists are very busy, and their time is valuable. Requesting an hour for an interview represents a loss of both time and income that could otherwise be spent seeing clients. Recruitment and finding participants willing to engage in this type of study were difficult. Additionally, many competing demands are placed on community therapists, in particular. Not only do they need to use time productively to see clients, but they must also complete paperwork and keep meticulous notes of each client session. This is also a population that is often asked to complete surveys about varying parts of their job or to help distribute survey information to colleagues. Therefore, this is a very difficult population to study with a representative, high participation rate. Future studies with this population are necessary, so studies should continue to consider therapists’ time limitations and schedules. Perhaps, gathering data at a continuing education event, or other event that therapists attend, but is not taking away from time for client care, would be a good way to reach this population in the future. A more representative sample of participants might also be obtained this way.

Moreover, the sample consists of only those who had their email or physical mailing (in the case of LMFTs) address available from a state or national database. Therefore, therapists in some states may not have received information about the study, and some states may be under-studied. Additionally, there was no way to track the response rate since it was not clear how many total potential participants received the study link. One reason for this limitation was funding. Many national lists and state databases require a fee in order to contact their membership. Some national databases cost upwards of $4,000, which was prohibitively expensive for this study. Other licensing agencies have a policy not to contact their membership from outside entities or for research purposes due to the number of requests they receive.

To better align research and clinical practice, stronger partnerships between researchers and clinicians are essential, particularly in the treatment of trauma. Disseminating research in accessible formats and supporting clinicians in tracking client outcomes can help bridge this gap. However, many therapists trained in graduate school may lack formal instruction or supervised practice in exposure therapy—especially *in vivo* exposure—due to limited graduate-level training. This lack of preparation may contribute to discomfort or underuse of exposure techniques in practice.

## Data Availability

The raw data supporting the conclusions of this article will be made available by the authors, without undue reservation.
